# Imaging of metabolic activity adaptations to UV stress, drugs and differentiation at cellular resolution in skin and skin equivalents – Implications for oxidative UV damage

**DOI:** 10.1016/j.redox.2020.101583

**Published:** 2020-07-19

**Authors:** Christopher Kremslehner, Anne Miller, Robert Nica, Ionela-Mariana Nagelreiter, Marie-Sophie Narzt, Bahar Golabi, Vera Vorstandlechner, Michael Mildner, Julia Lachner, Erwin Tschachler, Francesca Ferrara, Kristaps Klavins, Markus Schosserer, Johannes Grillari, Arvand Haschemi, Florian Gruber

**Affiliations:** a Department of Dermatology Medical University of Vienna, Austria; b Christian Doppler Laboratory for Biotechnology of Skin Aging, Austria; c Department of Laboratory Medicine Medical University of Vienna, Austria; d TissueGnostics GmbH, Vienna, Austria; e Division of Thoracic Surgery, Medical University of Vienna, Vienna, Austria; f Aposcience AG, Vienna, Austria; g Department of Life Sciences and Biotechnology, University of Ferrara, Ferrara, Italy; h Plants for Human Health Institute, North Carolina State University, Kannapolis, NC, USA; i CeMM Research Centre for Molecular Medicine of the Austrian Academy of Sciences, Vienna, Austria; j University of Natural Resources and Life Sciences,Vienna, Austria; k Ludwig Boltzmann Institute for Experimental and Clinical Traumatology, Austria

## Abstract

The epidermis is a multi-layered epithelium that consists mainly of keratinocytes which proliferate in its basal layer and then differentiate to form the stratum corneum, the skin's ultimate barrier to the environment. During differentiation keratinocyte function, chemical composition, physical properties, metabolism and secretion are profoundly changed. Extrinsic or intrinsic stressors, like ultraviolet (UV) radiation thus may differently affect the epidermal keratinocytes, depending on differentiation stage. Exposure to UV elicits the DNA damage responses, activation of pathways which detoxify or repair damage or induction of programmed cell death when the damage was irreparable. Recently, rapid diversion of glucose flux into the pentose phosphate pathway (PPP) was discovered as additional mechanism by which cells rapidly generate reduction equivalents and precursors for nucleotides – both being in demand after UV damage. There is however little known about the correlation of such metabolic activity with differentiation state, cell damage and tissue localization of epidermal cells. We developed a method to correlate the activity of G6PD, the first and rate-limiting enzyme of this metabolic UV response, at cellular resolution to cell type, differentiation state, and cell damage in human skin and in organotypic reconstructed epidermis. We thereby could verify rapid activation of G6PD as an immediate UVB response not only in basal but also in differentiating epidermal keratinocytes and found increased activity in cells which initiated DNA damage responses. When keratinocytes had been UVB irradiated before organotypic culture, their distribution within the skin equivalent was abnormal and the G6PD activity was reduced compared to neighboring cells. Finally, we found that the anti-diabetic and potential anti-aging drug metformin strongly induced G6PD activity throughout reconstructed epidermis. Activation of the protective pentose phosphate pathway may be useful to enhance the skin's antioxidant defense systems and DNA damage repair capacity on demand.

## Highlights

• Automated whole tissue histocytometry localizes enzymatic activity of G6PD within the epidermis at cell resolution.
• Keratinocytes in the tissue context react to UVB radiation with immediate activation of G6PD.
• Highest induction of G6PD was observed in cells undergoing DNA damage repair.
• The drug Metformin strongly induces epidermal G6PD activity.

## Graphical abstract

**Figure:**
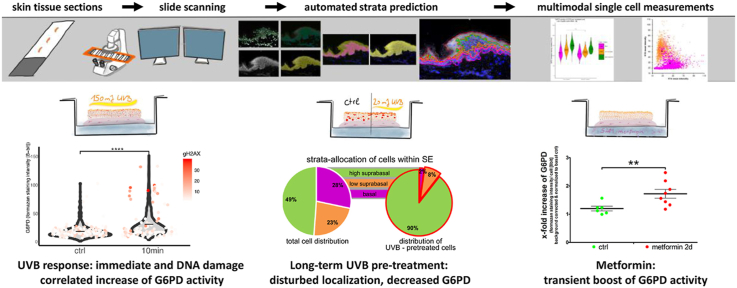

## 1 Introduction

The human skin is a complex three dimensional organ consisting of an outer epidermal and an inner dermal layer. Additionally skin contains appendages such as hair follicles or sebaceous glands. The epidermis is a multi-layered epithelium that consists mainly of keratinocytes (KC) as well as dendritic epidermal Langerhans cells and melanocytes. The KC proliferate in a basal layer where they are attached to the basement membrane, but as soon as they detach, stop proliferation and undergo a terminal differentiation program. Thereby they form a continuously renewing dynamic barrier to the environment [1]. This transformation process goes in hand with massive changes in the organelle- and macromolecular composition. Both the proteome and the lipidome are adapted to produce the components of the epidermal permeability barrier [2,3], and the nuclei and nucleic acids are degraded [4]. Differentiation is correlated with the accumulation of superoxide anions [5] and other mitochondrial reactive oxygen species [6]. There is a differentiation dependent gradient in constitutive and inducible redox regulatory networks that protect the epidermis from oxidative damage [7]. The ability of cells within the skin to protect from- or adapt to environmental stressors is dependent on cell type and –ontogeny, on the differentiation- and stress response state and on age [[8], [9], [10]].

Recently a novel type of keratinocyte (and fibroblast) oxidative stress response to ultraviolet radiation (UV)- or hydrogen peroxide exposure was described. It utilizes rapid metabolic stress adaptation through re-routing of glucose catabolism to the pentose phosphate pathway (PPP) and thereby generates reducing equivalents (NADPH) and nucleotide precursors [11]. Glucose 6-Phosphate Dehydrogenase (G6PD) is the first step for metabolizing glucose through the oxidative branch of the PPP, and can be rapidly activated for this task [12,13]. The products of PPP are in high demand in cells that have previously undergone exposure to UV-induced redox stress and nucleotide damage. The work that uncovered this mechanism was performed in keratinocytes and fibroblast grown in cell culture, thus there was a need to study this connection of metabolism and stress response in the organ context of the skin.

Glycolysis and oxidative phosphorylation (OXPHOS) are the main source of cellular energy (ATP), and the glycolytic end product pyruvate is either metabolized through the citrate cycle and respiratory chain or fermented to lactate or ethanol. Electron microscopy studies of the human epidermis suggested that only epidermal keratinocytes of the basal layer had mitochondria able to perform OXPHOS. Also, the epidermis produces large amounts of lactate irrespective of oxygen presence, and thus epidermal metabolism was regarded functionally anaerobic [14]. This was supported by the findings that the loss of the functional electron transport chain (ETC) did not impair epidermal stratification and barrier formation in the mouse [15]. In the basal KC however, as in other tissues with rapid proliferation [16] one could expect a need for NADPH to provide the reducing power to cope with superoxide generated during OXPHOS [17]. A role for G6PD in the epidermis as an enzyme that produces NADPH, which then serves as substrate for glutathione reductase and thereby an important antioxidant of the skin has long been recognized [18,19]. Thus it was of relevance to localize in cellular resolution the activity of G6PD and to address the metabolic and antioxidant functions that depend on position and differentiation state. The challenges in the study of metabolism in permanently regenerating stratified epithelia have been described early by Harris and colleagues [20] which demonstrated how starvation reduced- and proliferation stimuli increased glycolysis in mouse epidermis, but could not localize the activity. We have recently presented a method to assess the metabolic configuration of single cells in complex human tissues [21]. This method utilized visualization and quantification of enzymatic activities at saturating substrate conditions together with subsequent cell type identification. We here combined this method with automated prediction of epidermal strata using combined histological bright-field and fluorescent image analysis. This allowed assessing enzymatic activity of G6PD at cellular resolution within specific epidermal strata. Furthermore, we utilized an organotypic reconstituted epidermis which was exposed UV-irradiation or the metabolic modulator Metformin. The UV exposure was either applied a proportion of the cells that were used to engineer the skin equivalent or UV was applied later to the already stratified epidermal equivalent. We could confirm that KC in the tissue context respond to UV radiation by upregulation of G6PD activity, and that this response was not confined to basal KC but also detectable in differentiating, suprabasal cells. Furthermore, we found that the response was increased in cells that had suffered DNA damage. KC that had been irradiated immediately prior to seeding within the epidermal equivalent showed an unexpected shift in their distribution within the epidermal equivalent towards the more differentiated strata, and a reduced activity of G6PD. Finally, exposure of the epidermal equivalents to Metformin led to a strong induction of G6PD activity, showing for the first time that this antidiabetic drug which is also being investigated as anti-ageing drug can regulate this key enzyme activity in a tissue.

## 2 Materials & methods

### 2.1 Primary keratinocyte culture, skin equivalent model and biopsies

Preparation of primary human keratinocytes (KC) was performed as previously described [22] from adult abdominal skin obtained from plastic surgery and cultured in serum-free keratinocyte growth medium (KGM-2, PromoCell, C-20211). Human full thickness skin equivalent models (SE) were generated as described by Ref. [23]. When indicated 20% pre-treated cells (UVB 20 mJ/cm^2^ or sham controls labelled with “Cell Tracker red” dye, see below) were included among untreated KC. The skin samples used for preparation of primary cells and for collection of biopsies for the cryosections used in this study was approved by the Ethics Committee of the Medical University of Vienna (1149/2016) and written informed consent was obtained from all subjects.

### 2.2 UVB treatment and fluorescent labelling

For UVB treatment primary cells were seeded in 6-well plates (Corning) (80% confluency) and growth medium was replaced with PBS for the duration of the irradiation. A total fluence of 20 mJ/cm^2^ at a distance of 20 cm was achieved using a Waldmann UVB source (F15 T8 tube, maximum emission at 318 nm, see also [24]), as measured with a Waldmann UV-meter (Waldmann, Germany). After irradiation treated cells were incubated with a fluorescent cell tracker (20 μM, CellTrackerTM Red CMTPX, Thermo Fischer Scientific) for 90 min under growth conditions. The labelled cells were allowed to recover for 1 h in fresh medium before they were mixed with un-treated cells and seeded into the skin equivalent models in KGM-2 medium. In order to investigate the immediate effects of UVB exposure on the skin equivalent model, the full-grown SE were irradiated with 150 mJ/cm^2^ and cryo-samples were taken one and 10 min after irradiation respectively.

### 2.3 Metformin treatment

Skin equivalent models were treated with one dose of 5 mM metformin in supplemented keratinocyte defined medium for 48 h, either at the introduction air-liquid interface (7 days before) or 2 days before cryo-sampling. For mass-spectrometric analysis of metabolites and measurement of glucose consumption normal human keratinocytes were grown in monolayer culture and treated with 5 mM metformin in KGM-2 once they reached confluence. Culture medium was sampled before treatment and after 24 h for glucose consumption and cells were subsequently washed with PBS and scraped with methanol for mass spec analysis.

### 2.4 Glucose-6-phosphate dehydrogenase activity assay

For the G6PD enzymatic activity assay non-fixed cryosections were stored at −80 °C at least overnight and were treated as described in Ref. [21]. The reaction was adapted as follows: Polyvinyl alcohol was omitted from the 0.1 M Tris-Maleate buffer pH 7.5 for better handling and DHEA concentration was reduced to 1 mM, which is close to the solubility limit in aqueous solutions but was still sufficient to inhibit the G6PD reaction. Further the cryosections were defrosted in pre-warmed moist chamber for 5 min and the subsequent enzyme activity staining was performed with pre-warmed buffer in a moist chamber at 37 °C protected from light.

### 2.5 Immunofluorescence staining

Immediately after the G6PD activity assay tissue sections were fixed with 4% para-formaldehyde for 10 min at room temperature and, in case of nuclear staining, were blocked and permeabilised with 0.5% (v/v) Tween-20 (Sigma), 1% (w/v) BSA (Sigma), 10% (w/v) gelatin from cold water fish skin (Sigma) in PBS (Gibco) for 30 min. The sections were incubated with the primary antibodies or without antibodies for background staining control in 2% (w/v) BSA in PBS overnight at 4 °C. Subsequently the sections were washed 3 times in PBS and stained with Hoechst reagent and ALEXA fluor 2nd antibodies (1:500) or directly labelled antibodies in 2% (w/v) BSA, 10% (v/v) specific anti serum in PBS at room temperature protected from light for 30 min. After additional 3 washing steps with PBS for 5 min each the sections were dried and mounted in Permafluor (Thermo Scientific).

### 2.6 Image acquisition and analysis

Tissue sections that had been subjected to the enzymatic activity assay and subsequent nucleic counterstaining with Hoechst reagent and immunofluorescence stainings were digitalized with a TissueFAXS I PLUS slide-scanning microscope (Zeiss Observer Z1, 12-slide stage, Hamamatsu Orca Flash 4.0 V3 4K, X-Cite Series 120PCQ Laser). Both brightfield images of the enzymatic-activity staining as well as immunofluorescence images were acquired with the same monochrome camera to guarantee perfect alignment of the different channels. As suggested by the manufacturer preview images of the nuclei staining were acquired with a 2.5x objective for region of interest selection. The acquisition was performed with a 20x objective using the corresponding filters for DAPI, FITC, Cy5, Texas Red or TL to detect selected fluorescence signals with exposure times and thresholds adjusted for each experiment and filter individually. Once the adjustments were set they were kept for all acquisitions of individual experiments. Images were acquired using the implemented auto-focus with extended focus options for fluorescence channels (selecting for each pixel the value with the highest contrast from two images above and two images below the focus point in 1 μm steps). The focus strategy applied used a floating focus point and focus interval was set around a manually defined z position.

The data set was imported into the StrataQuest image analysis software (version 5.0.1.336) and processed with a customized analysis profile to predict the epidermis, its different strata and single cell measurement masks, developed from the initially described method by Ref. [25].

For the prediction of the epidermis two seed masks were generated, the first based on a density map of all nuclei in the section detected by the StrataQuest “nuclear segmentation” engine (version2) and the second on a thresholded version of the inverted brightfield image, utilizing the differences between epidermis and dermis concerning both the nuclei density and the optical density of the two compartments respectively. After manual correction of the epidermis prediction the distinction between the basal layer and the stratum corneum was based on the measurement of area per nuclei. The prediction of the stratum corneum was defined as the contour section of the epidermis containing the lower fraction of nuclei and the prediction mask was expanded to the boarder of the nucleated area as established by the nuclei density map. Starting from the nuclei denser part of the epidermal contour the basal layer was defined on a distance-based manner to only include the first layer of epidermal nuclei and cell-masks. The same procedure was repeated to obtain the first suprabasal layer with the only difference being that the basal layer was used as the starting point.

In order to enable measurements on a single-cell level cellular prediction masks were grown within the basal, low suprabasal and suprabasal strata and a maximal radius of 10 μm, utilizing the StrataQuest “cellular mask” engine (version 2) and the detected nuclei as seeds.

### 2.7 Mass spectrometry - metabolomics

The liquid/liquid extraction using mixtures of water, methanol, and chloroform was utilized for the metabolite extraction from cell culture samples. First, 10 μl of isotopically labelled internal standards was added to samples followed by addition of 450 μl of methanol. Samples were vortexed for 15 s and 450 μl of chloroform was added and again vortexed for 15 s. Afterwards, 140 μl of water was added, samples were vortexed for 10 s, incubated on ice and vortexed again for 10 s. Sample were centrifuges for 10 min at 100 g, the upper aqueous phases was collected and dried downs using nitrogen evaporator. Samples were reconstituted in 50 μl of water and used for LC-MS/MS analysis. An Acquity UHPLC system (Waters) coupled with a Xevo TQ-MS triple quadrupole mass spectrometer was used for LC-MS/MS analysis. The chromatographic separation for samples was carried out on an ACQUITY HSS T3, 1.8 μm, 2.1 × 100 mm analytical column (Waters) equipped with a VanGuard BEH C18, 2.1 × 5mm pre-column (Waters). The column was maintained at a temperature of 40 °C and 2 μl sample were injected per run. The mobile phase A was 0.1% formic acid (v/v) in water and mobile phase B was 0.1% formic acid (v/v) in methanol. The gradient elution with a flow rate 0.5 mL/min was performed with a total analysis time of 10 min. The mass spectrometer was operated in positive and negative ionization mode, capillary voltage 3.2 kV, desolvation temperature 550 °C, desolvation gas flow 1000 l/h, cone gas flow 100 l/h. The multi reaction monitoring (MRM) mode was used for the detection of metabolites. The MassLynx V4.2 software (Waters) was used for the data processing. Seven-point linear calibration curve with internal standardization was employed for the quantification of metabolites.

### 2.8 Glucose consumption

Cells were cultured in KGM2 (PromoCell, C-20211) for 24 h. At the timepoint of collection, the supernatant was centrifuged at 18000g for 20 min and then precipitated with the fourfold volume of MeOH and vortexed rigorously. The samples were again centrifuged at 18000g for 20 min, the supernatant was transferred into a new tube and desiccated. The metabolite pellets were resuspended in type 1 water (18.2 MΩ-cm). The samples were analyzed using high performance anion-exchange chromatography with pulsed amperometric detection using a Dionex ICS-5000+ equipped with a PA20 analytical column and a Gold Ag–Ag Cl Carbo, Quad detector (Thermo Fisher). The eluent was sodium hydroxide in type 1 water (18.2 MΩ-cm) in isocratic flow conditions with a flow rate of 0.4 mL/min and a column temperature of 30 °C. The quantitative analysis of the glucose peak areas was based on measuring standards and subsequent calibration curves.

### 2.9 Statistical analysis and graphs

Single cell measurements of the enzyme activity staining were exported from StrataQuest to Microsoft Excel 2010. After formatting the raw imaging data sets were loaded into R version 3.6.2 (2019-12-12) -- “Dark and Stormy Night” (The R foundation for statistical computing, Vienna, Austria). Graphs were created using ggplot2 version 3.2.1. in R, or in GraphPad Prism 5 software. The statistical significance of differences was calculated with unpaired 2-tailed Student's t-test if not indicated otherwise and significances are indicated in the figures by asterisk (*p < 0.5, **p < 0.1, ***p < 0.001, ****p < 0.0001).

## 3 Results

### 3.1 Prediction of epidermal strata and shapes of residing cells using an image analysis algorithm applied on composite IF and brightfield images

**Fig. 1:**
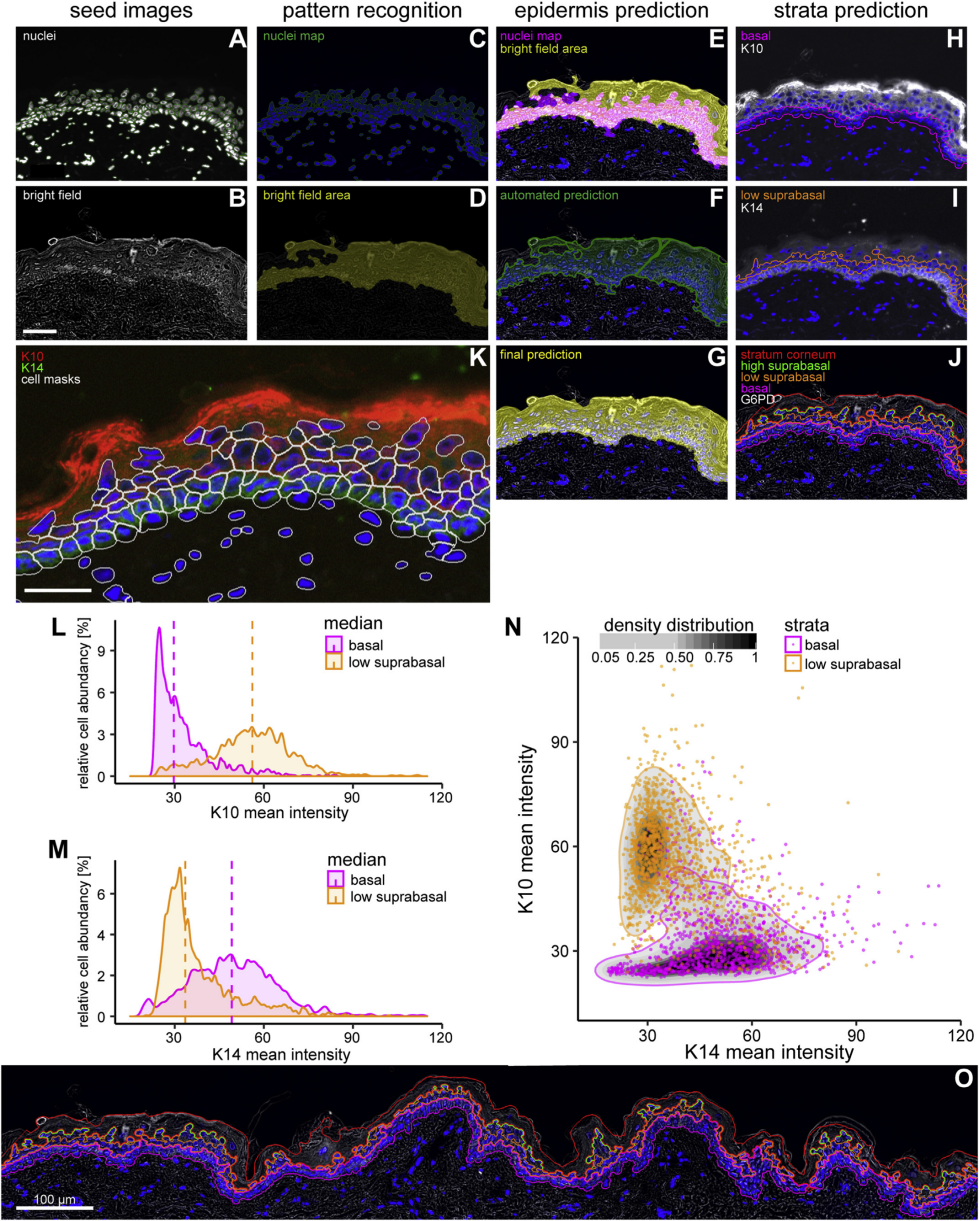
Workflow of epidermis recognition and counter-staining based validation of strata prediction. Cryosections of human skin biopsies were stained by immunofluorescence for the keratinocyte differentiation markers keratin 10 (K10, only expressed in suprabasal, differentiating KC), keratin 14 (K14, highly expressed in basal KC) and nuclei were stained with Hoechst dye. Whole tissue sections were scanned in fluorescent and brightfield modalities with a TissueFAXS slide scanning microscope at 20-fold magnification and subsequently processed with “StrataQuest” image analysis software. (A) Hoechst reagent stained human abdominal skin section and (B) brightfield micrographs were used as seed images for tissue prediction with StrataQuest software. Using the nuclear fluorescence channel, nuclei were automatically detected. (A, green contours). The engine “Label Graph from Coded v3” was used to connect nuclei that are in close proximity, empty spaces between the nuclei in proximity were filled using the “Morphological – BWFillHoles” engine and “nuclei map” image (C) was generated using the “Morphological – BWAreaOpen” engine. On the brightfield (“Tran channel”) image (B), a “Filter Gauss” engine was used and thresholded (“Total Area Measurements” engine). Holes were filled (“Morphological – BWFillHoles” and “Morphological – close (dilate, erode)” engines), small clusters were removed (“Morphological – BWAreaOpen” engine) resulting in “bright field area” (D). The “nuclei map” was used as a seed and grown on the “bright field area” image (E). The resulting image is the “automated prediction” (F) of the epidermis. Manual correction was applied to correct the automatic process for the “final prediction image” (G). The basal stratum (H, pink contour) was grown on the nuclei-denser edge of the epidermis based on its distance from the border. The same growth step was applied for the low suprabasal strata (I, orange contour) with the basal layer as starting point. The stratum corneum (J, red contour) was grown from the edge of the outside of the epidermis down to the edge of the “nuclei density” map. The high suprabasal strata (J, green contour) were added between the stratum corneum and the low suprabasal stratum. (K–N) IF staining for keratin 10 (K10 stained in white in H, red in K) and for keratin 14 (K14 stained white in I, green in K). Histogram presentations of abundance vs staining intensity of the predicted cells positive for K10 (L) and K14 (M) in the respective predicted strata. The median of the individual populations is indicated by dashed vertical lines. (N) Scatter plot (K10/K14) of predicted cells in basal (pink) and low suprabasal (orange) strata. The density of the different point clouds is indicated by the greyscale color bar and the outlines delimit the 95% density distributions of the respective strata. (O) Epidermal and strata prediction on a continuous stretch of a representative normal human skin tissue section (Size bars: 50 μm, except in O: 100 μm).

A goal of this study was to correlate (immuno-) histological parameters for identity and enzymatic activity of single cells to their position within the skin or the epidermis and to their differentiation state. To achieve this we needed to assign cells to the skin compartments and to their position within these compartments. We chose an automatized and high-throughput approach termed “automated whole tissue histocytometry” to achieve this assignment on a large number of cells within the human epidermis. The human epidermis has features which allow the human eye, and image analysis algorithms, to recognize its delimitations without the need for immunostaining. Firstly, the tight linear arrangement of nucleated basal KC along the basement membrane serves as a reliable guidance feature to find the innermost delimitation of the epidermis in sections that are stained with a nuclear dye. Secondly, the differentiation of the keratinocytes yields suprabasal spinous, granular and cornified strata which yield a characteristic contrast pattern visible in brightfield – and phase contrast microscopy and indicate the uppermost delimitation of the skin. Sections of human skin were scanned in fluorescent (DAPI channel) and brightflield modes to generate seed images (Fig. 1 A, B). On the seed images the “Label Graph from Coded v3”, “Morphological – BWFillHoles”, “Morphological – BWAreaOpen”, “Total Area Measurements” and “Morphological – close (dilate, erode)” features of the StrataQuest software were applied to generate maps of nuclei (Fig. 1C) and areas of higher contrast (Fig. 1 D). The overlaid maps (Fig. 1 E) were used to predict the epidermal area (Fig. 1 F). This area received manual correction only in case of histological preparation artefacts (e.g. folded tissue, Fig. 1 G). The distance mapping of the nuclei was used to predict the basal layer of the epidermis (Fig. 1H). Based on this basal layer the software predicted the next peripherally predicted row of cells as „low suprabasal layer“ (Fig. 1 I). The area between the outside border of the brightfield area and the first nucleated cells was used to predict the stratum corneum, and the area between the stratum corneum and the “low suprabasal” layer was classified as “high suprabasal” layer (Fig. 1 J), completing the strata prediction. Furthermore, we used “Build Measurements Mask – Cellular” feature of StrataQuest to predict cell boundaries and the area of the cytoplasm. This algorithm uses the boundaries of the nuclear area to grow the predicted cell shapes (Fig. 1 K, white lines).

Next, we investigated how the predicted strata and the predicted cells within the defined area would correspond to protein marker expression expected for the strata they had been allocated to. Undifferentiated basal keratinocytes express keratins 14 and 5, but when they differentiate and move toward the spinous layer, then the granular layer and ultimately the stratum corneum they express the keratins 1, 2 and 10, among other differentiation associated proteins (K10 stained in white in Fig. 1H, red in Fig. 1 K; K14 stained white in Fig. 1 I, green in Fig. 1 K). The StrataQuest software allows to present the parameters of the single predicted cells in the form usual in flow cytometry. We used these histogram features to plot the mean immunofluorescent staining intensities for K10 (Fig. 1 L) and K14 (Fig. 1 M) of the cells allocated to the basal (pink) and suprabasal strata (orange), and found two clearly distinct populations of keratinocytes. These, also presented as a K10/K14 scatter plot of predicted cells represent the nucleated undifferentiated basal and nucleated differentiated suprabasal keratinocytes (Fig. 1 N).

From those predicted cells masks (cytoplasms) located in the predicted basal stratum 5 to 7% of the predicted cells were negative for K14 expression (cells at the basal layer without green staining in Fig. 1K) as it can be expected that the basal layer contains on average 8% of nucleated non-epithelial cells, mostly melanocytes [26]. Once established the algorithm was applied to large tissue sections as exemplified in (Fig. 1 O) in order to generate data sets with large numbers of quantified events for the further experiments.

We conclude that the prediction with StrataQuest software allowed for sufficient distinction between suprabasal differentiated and non-differentiated basal epidermal keratinocytes. This allows addressing scientific questions that gain from this distinction and need to be solved at cellular resolution. Further, this label free method allowed defining a low- and a high suprabasal stratum in which cell shapes for localized multimodal measurements can be predicted, as well as the area of the stratum corneum, all of which can be investigated using various modalities provided by the imaging system.

### 3.2 A tetrazolimum salt-based enzymatic activity assay in combination with automated tissue histocytometry can spatially resolve activity of G6PD in skin and in organotypic epidermal equivalents

**Fig. 2:**
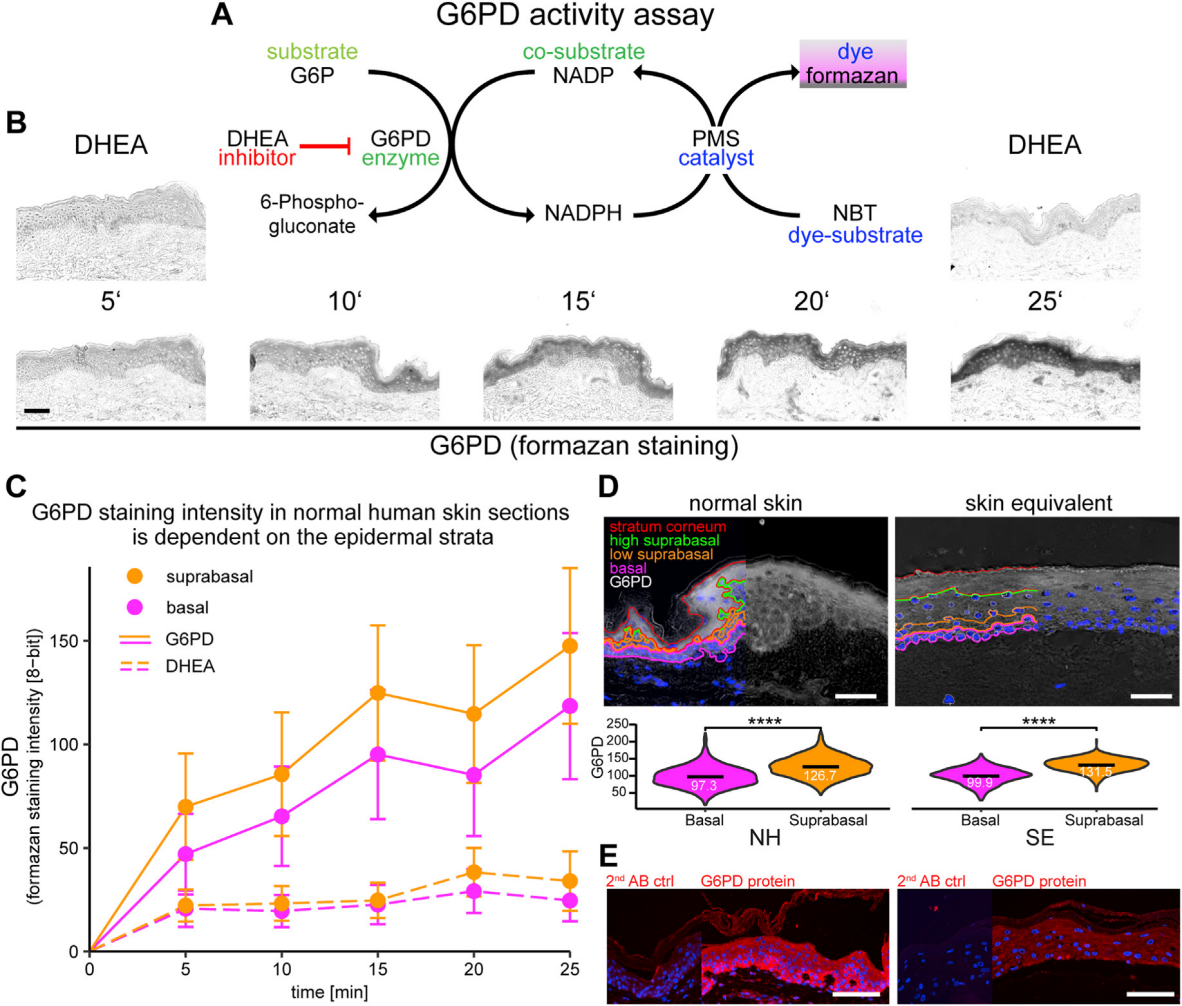
Formazan-based enzymatic activity assay of G6PD in skin and equivalence of activity distribution between reconstructed epidermal equivalents and normal human skin. Non-fixed cryosections of normal human skin were subjected to a formazan-based enzymatic activity assay for glucose-6-phoshate dehydrogenase (G6PD). (A) Schematic mode of action of the enzymatic activity assay. The electron released during the conversion of G6P by the tissue native enzyme G6PD is shuttled via the electron carrier phenazinium methylsulfate (PMS) to nitroblue tetrazolium chloride (NBT) that in turn forms a non-soluble formazan salt. The reaction was stopped by washing at the indicate time points. The non-competitive selective inhibitor of G6PD dehydroepiandrosterone (DHEA, 1 mM) was used as a negative control. (B) Representative brightfield images of cryosections subjected to the G6PD activity assay at 5 indicated time-points. Cryosections exposed for the indicated timepoints to which DHEA had been added are shown as negative controls for the start (5′) and end (25′) time points (upper panels). (C) Quantification of the increase in G6PD enzymatic activity staining in basal and suprabasal strata of 3 consecutive sections from normal human skin biopsies from 3 donors over time. The dashed line indicates the G6PD activity measured in the sections where the assay reaction was inhibited by addition of DHEA. (D) Top panel: Comparison of G6PD enzymatic activity staining and strata prediction in normal human skin (left) and reconstructed epidermal equivalents (right). Lower panel: Violin plots of cellular G6PD activity distribution in the basal and the suprabasal strata in normal human skin and SE. (E) IF staining for G6PD protein (bottom). Staining control performed with secondary antibody only (narrow sections). For D the mean values are indicated by black bars with subscribed numeric values. Asterisks indicate statistical differences (****- p < 0.0001; Student's t-test calculated with R software). Size bars: 50 μm.

G6PD is the rate-limiting enzyme for glucose to enter the PPP and is involved in nucleotide biosynthesis and in providing NADPH. To map in high resolution the activity distribution of this enzyme in skin we adapted the method described in Miller et al. [21] to combine automated whole tissue histocytometry with enzyme histochemistry for G6PD on cryosections of human skin and on human skin equivalents. For the enzyme histochemistry protocol [27] nitroblue tetrazolium chloride (NBT) was used as a detection reagent. This colorless tetrazolium salt yields a colored formazan precipitate when reduced by NADPH/H^+^, the co-factor product of G6PD, in the presence of the electron carrier 1-methoxy phenazinium methylsulfate (PMS). Specificity and reproducibility of this assay in frozen sections of various tissues have been described in Ref. [21], and we performed time response curves to confirm the linear detection range of this assay in skin sections scanned in brightfield mode (reaction principle scheme in Fig. 2 A). Similar to what we had observed in liver and muscle [21], the formation of the formazan precipitate was time dependent and mostly linear in a reaction timecourse up to 25 min (Fig. 2 B, C), and could be inhibited with the non-competitive G6PD inhibitor dehydroepiandrosterone (DHEA, 1 mM) at the indicated time-points (Fig. 2 B top panels left and right). The time-dependent increase of enzymatic activity staining was confirmed in two additional donors (data not shown). The activity of G6PD, defined by formazan staining intensity, was highest in the granular layer of the epidermis, but inhibitable activity was observed throughout the epidermis (Fig. 2D top left), in line with earlier findings [28]. Next, we applied the combined histocytometric and enzyme histochemistry method to the *in vitro* organotypic skin equivalent model we have refined [23]. This skin equivalent model allows modeling a fully stratified epidermis which develops within seven days when primary human keratinocytes seeded on top of a dermal equivalent layer are exposed to an air-liquid interface in culture inserts. The epidermis is in many aspects equivalent or highly similar to native human epidermis. This was shown for gene expression, lipid composition, the translation and processing of barrier proteins of the keratinocytes and barrier function [22,29,30]. We found that also the activity of G6PD within the skin equivalent was comparable to human skin, with maximum activity being observed in the suprabasal and granular layer (Fig. 2D top right panel). The violin plots show comparable cellular activity distributions of G6PD in epidermis and SE, with significantly higher activity in suprabasal as compared to basal cells (Fig. 2D lower panels). Interestingly, the epidermal distribution of the G6PD protein (or the accessible epitope) as detectable with an antibody, differed from the distribution of its activity. In both, skin biopsies and skin equivalents, the protein could be detected throughout the epidermis, but the strongest signal was observed in the basal epidermal layer (Fig. 2E). This observation suggests that KC differentiation-related factors affect the activity of G6PD.

3.3 Exposure of the skin equivalent to UVB resulted in immediate elevation of G6PD activity in all strata of the epidermal equivalent, most strongly in cells undergoing DNA damage repair.

**Fig. 3:**
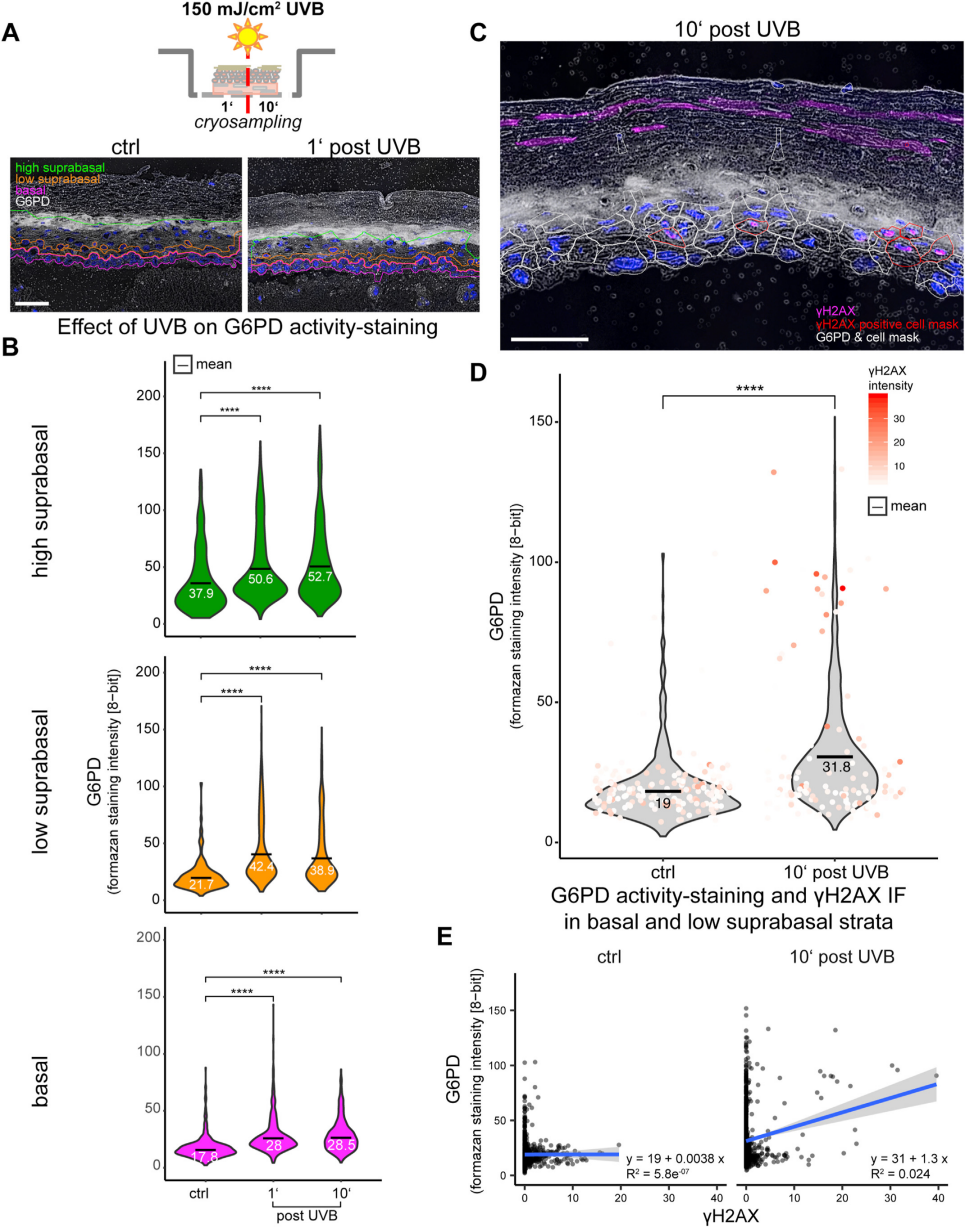
UVB exposure of human epidermal skin equivalent models leads to immediate increase of G6PD activity staining. Fully stratified human epidermal skin equivalents were irradiated with UVB (150 mJ / cm²) and cryo-samples were collected one and 10 minutes after end of irradiation (A, top scheme). Shown are representative graphs from 3 independent experiments. (A) Lower panels: Inverted brigh-tfield images of G6PD activity staining in cryosections from control skin equivalents and one minute post irradiation. Basal and low suprabasal strata indicated by pink and orange contour respectively. (B) Violin plots of G6PD activity staining at the indicated time points within the indicated strata. (C) Inverted bright-field image of cryosections from skin equivalents 10 minutes post irradiation with overlaid IF staining for γH2AX. White outlines indicate predicted cellular measurement masks, cell masks of γH2AX positive cells are marked by red contours. D) Violin plots of G6PD activity staining in the combined basal and low suprabasal strata of control samples and samples taken 10 minutes post irradiation to which values of γH2AX positive cells are superimposed, the color gradient indicating relative IF staining intensity. (E) Scatter plots of G6PD activity staining in the combined basal and low suprabasal strata of control samples and samples taken 10 minutes post irradiation versus γH2AX-staining intensity with regression lines (gray: 95% CI). For B and D the mean values are indicated by black bars with subscripted numeric values. Asterisks indicate statistical differences (****- p<0.0001; Student’s *t*-test calculated with R software). Size bars: 50µm.

Next, we investigated whether and where within the skin equivalents exposure to UVB radiation would modulate the G6PD activity. To this end we exposed the skin equivalents to 150 mJ/cm^2^ of UVB and collected samples 1 min and 10 min after end of irradiation (Scheme Fig. 3 A, top). In Fig. 3 A (bottom panels) typical inverted G6PD micrographs are shown on which the prediction of the strata was superimposed. Histocytometric analysis of activity in cells attributed to the basal, and suprabasal strata showed that G6PD activity was significantly increased as early as 1 min after irradiation. Whereas the highest absolute response was observed in the low suprabasal strata from 1 min post exposure onwards (violin plots of mean cellular formazan staining intensity shown in Fig. 3 B middle panel, orange), significantly increased activity was also observed in the basal layer (Fig. 3 B bottom panel, pink) and the high suprabasal layers (Fig. 3 B top panel, green), despite the higher baseline activity observed in the latter. This suggests that despite the presence of a strong differentiation dependent activity gradient for this enzyme, there likely is an independent mechanism for UV-mediated G6PD activation.

Phosphorylation of H2AX is rapidly induced upon UV induced DNA damage and correlated with nucleotide excision repair (NER) also in non-dividing cells [31,32]. The DNA damage response is enhanced by activation of the PPP, and supplementation of its products NADPH and ribose can promote DNA damage repair [33]. Functional G6PD is required to reduce γH2AX foci in cells that had undergone gamma irradiation [34]. Thus we next assessed whether the induction of G6PD activity could be correlated to UVB-induced DNA damage. We used automated histocytometry to quantify presence and intensity of γH2AX immunostaining in nuclei, and G6PD activity in the predicted cytoplams 10 min after irradiation. In Fig. 3C the predicted cell shapes of cells with γH2AX-positive nuclei in a UVB-irradiated sample are highlighted in red. The results are depicted as overlay of a violin plot of G6PD activity in the combined basal and low suprabasal strata with a jitter plot that color-codes the nuclear intensity of γH2AX from white (low) to high (red) (Fig. 3D). It shows that most events (predicted cells) with high UV-induced nuclear γH2AX immunostaining map to the group of cells with high G6PD activity. The regression analysis in the scatter plots show that upon UVB irradiation, there was positive correlation of γH2AX staining intensity with G6PD activity (Fig. 3 E). These findings are compatible with the notion of DNA damage-inducible activation of PPP, but the speed of activation suggests that this mechanism is independent from classical DDR responses with transcriptional regulation of PPP [35].

### 3.3 KC that had been irradiated with UVB prior to seeding into the undifferentiated skin equivalent display significantly reduced G6PD activity and aberrant distribution within the epidermis

**Fig. 4:**
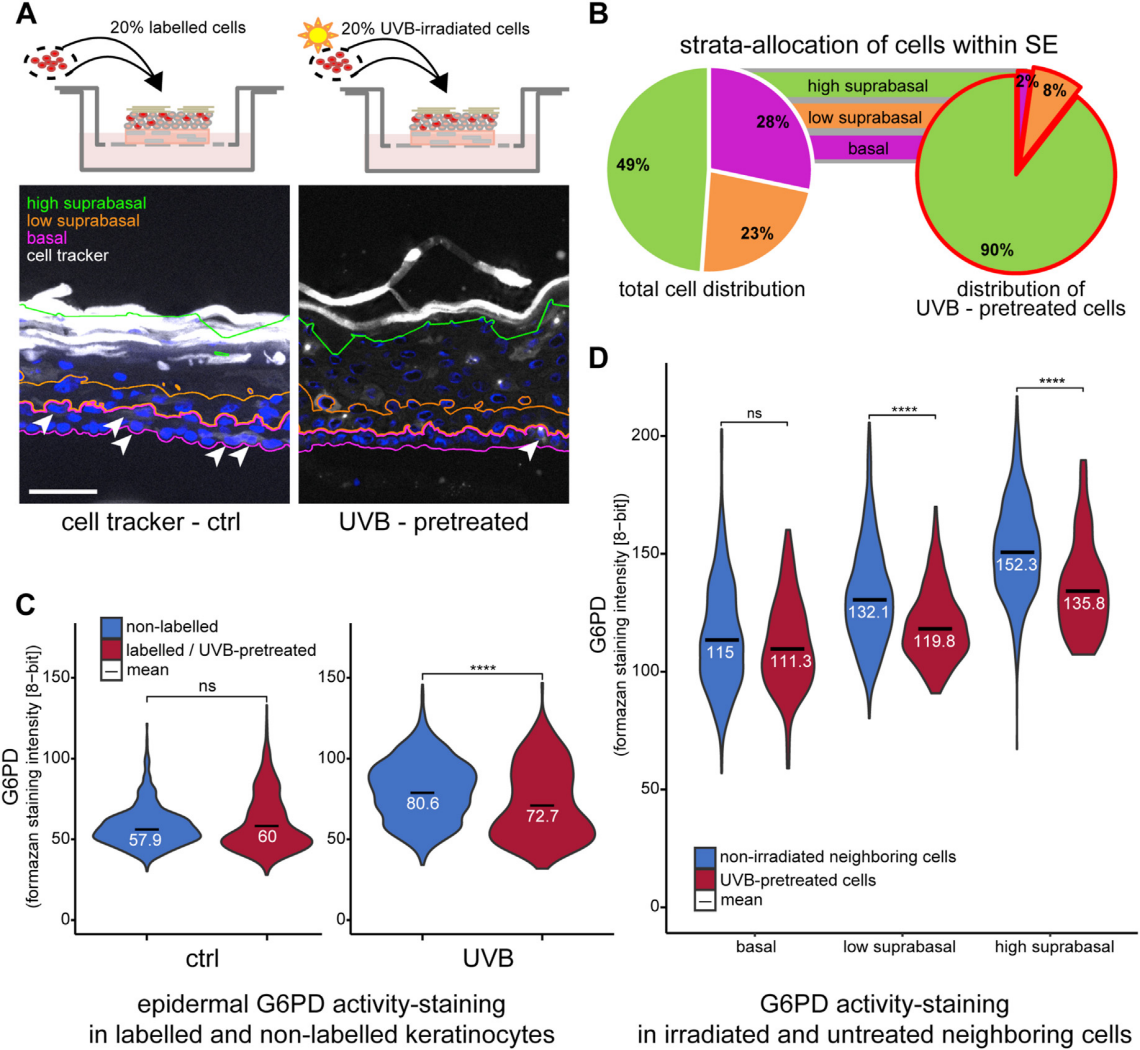
UVB pre-treatment effects the location of keratinocytes within the skin equivalent model. Skin equivalent models were generated with 20% labelled or UVB pre-treated labelled cells and their location within the different strata of the fully differentiated skin equivalent model as well as their G6PD activity were assessed. A) IF image of cell tracker (white) labelled keratinocytes within the skin equivalent model, basal, low & high suprabasal strata indicated by pink, orange and green contours respectively. Labelled basal cells are indicated by arrow heads. B) Pie chart depicting the distribution of all cells to the epidermal strata and the distribution of UVB pre-treated, labelled cells to the strata (red contour pie chart). C) Violin plots of G6PD activity staining. Left: activity plot of non-labelled cells (blue) in comparison to labelled untreated cells (red) within the same skin equivalent. Right: activity plot of non-labelled cells (blue) and labelled UVB pre-treated cells within the same skin equivalent. D) Violin plots of G6PD activity staining of UVB pre-treated labelled cells (red) and their non-irradiated neighboring cells (blue) within the different strata of the skin equivalent model. For C and D the mean values are indicated by black bars with subscripted numeric values. Asterisks indicate statistical differences (****p < 0.0001; ns-no significant difference; Student's t-test calculated with R).

To investigate, whether the metabolic response would be sustained for a longer period post irradiation, we pre-irradiated and then dye-labelled 20% of the keratinocytes before seeding them into the skin equivalent (Scheme Fig. 4 A, top). We investigated the G6PD activity of the labelled cells when the fully stratified skin equivalent was harvested seven days after air-liquid interface. We observed that the irradiated cells were not distributed in the expected pattern throughout the skin equivalent as pre-irradiated cells were virtually absent from the basal layer (Fig. 4A and B). This finding is compatible with other observations that KC which undergo DNA damage signaling [36,37], or cellular senescence [38] lose their ability to attach to the basement membrane or are preferentially removed via differentiation, as proposed by others [39]. While the non-irradiated keratinocytes displayed a comparable distribution of G6PD activity compared to the surrounding unlabeled cells (Fig. 4C. left violin plots), a significantly lower activity was observed in the pre-irradiated labelled KC compared to non-irradiated cells within the same tissue (Fig. 4C, right violin plots). The finding that the cells that had received UVB 8 days prior to analysis but were still nucleated and neither fully differentiated nor apoptotic, showed reduced G6PD activity, was unexpected. A reduction in G6PD in KC has been reported for keratinocytes from aged donors in comparison to those from young donors [40], so further studies will have to show whether this metabolic configuration is typical for (photo) ageing of KC at the cellular level within the tissue.

### 3.4 Metformin strongly induces G6PD activity in undifferentiated and differentiated keratinocytes and throughout the epidermal equivalent

**Fig. 5:**
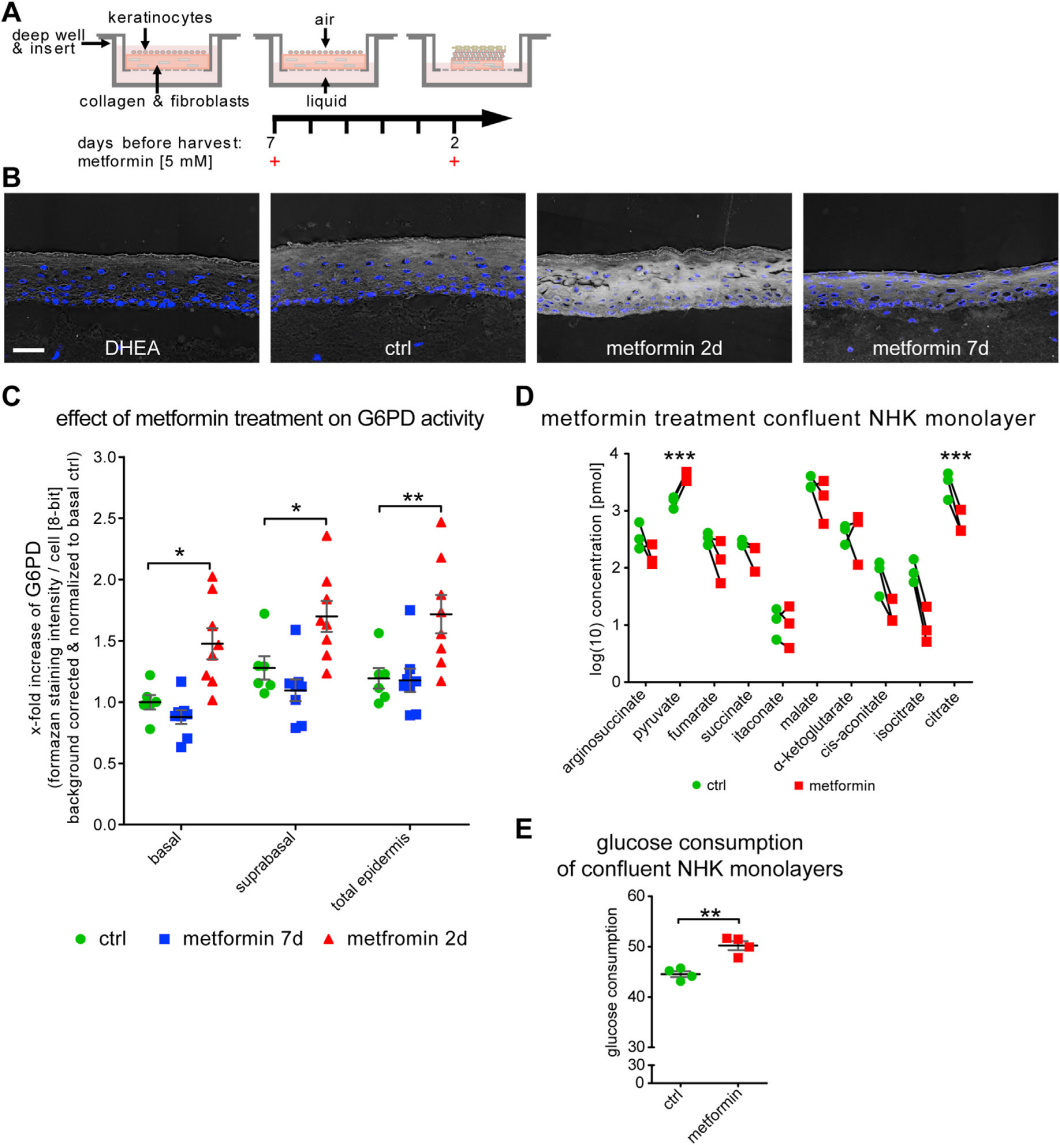
Treatment with metformin transiently increased G6PD activity staining in human skin equivalent models. Human skin equivalent models were treated with 5 mM metformin either at the onset of differentiation (metformin 7d) or 2 days before cryo-sampling (metformin 2d) and G6PD activity staining was assessed. Data from multiple consecutive cryosections from duplicate skin equivalent models generated with cells from three different donors. (A) Schematic of the skin equivalent metformin treatment regime. (B) Representative inverted bright-field images of G6PD activity staining in cryosections as indicated. (C) Metformin effect quantification for the indicated strata. Measurements from 3 independent experiments, mean values and SEM are indicated by the black bar and grey whiskers, respectively. (D) Change of metabolite concentrations in keratinocytes from 3 different donors grown as confluent monolayer cultures 24 h post metformin treatment determined with mass spectrometric metabolomics. (E) 24-h glucose uptake of keratinocytes grown as confluent monolayer culture (n = 4). Mean values and SD are indicated by back bar and grey whiskers respectively. Asterisks indicate statistical differences (*p < 0.05, **p < 0.01, ***p < 0.001; no labeling indicates no significant difference between ctrl and metformin groups; 2 way ANOVA with Bonferroni correction for (C) and un-paired 2 tailed Student's t-test for (D) were calculated with GraphPad Prism 5.) Size bar: 50 μm.

Next, we wanted to test whether we could activate G6PD pharmacologically and in the absence of actual UV induced damage. The commonly used anti-diabetic drug metformin has been identified as an inhibitor of UVB-induced skin tumorigenesis [41]. Thus we tested metformin for its ability to modulate G6PD activity in keratinocytes and the skin equivalents. We supplied the drug (5 mM) either at the beginning of the air-liquid interface period of culture (and thus at the onset of KC differentiation seven days before sampling) or two days before sampling (scheme Fig. 5 A). We found that in those skin equivalents that had been exposed to metformin two days prior to sampling, a very strong and significant induction of G6PD activity was observable throughout the SE (Fig. 5B and C). This effect was not observed in those SE that had been treated before onset of differentiation (metformin 7d) but these displayed mild parakeratosis (nucleated cells in the stratum corneum).

To get a further understanding on the regulation of glucose metabolism induced by metformin, we analyzed the glucose consumption in the supernatant of confluent primary KC (which served as model for basal undifferentiated KC in the skin equivalent) and revealed that 5 mM of metformin increased the glucose consumption by twelve percent. We also investigated the downstream fate of glucose in these cells and analyzed pyruvate and selected TCA cycle metabolites in confluent primary KC using mass spectrometry metabolomics. This revealed that metformin induced significant changes in the steady-state levels of several of the detected metabolites. In line with the observed induction of glucose consumption in these cells, we detected an accumulation of the glycolytic end-product pyruvate, indicating increased glucose metabolism in these cells. Interestingly, the pool size of most TCA cycle intermediates was decreased, most notably we detected a significant decrease in citrate levels. A possible explanation would be a further enhancement of the high lactate secretion observed in differentiated KC [14].

## 4 Discussion

The utilization of metabolic adaptations by cells to respond to genotoxic or oxidative damage is a novel and emerging field [11,40] and first mechanistic insights how this response is coupled to DNA damage repair have been reported recently [42]. Earlier studies on cellular metabolism investigated cellular oxygen consumption, -acidification or the activities of metabolic key enzymes on cultured cells, their supernatants or tissue homogenates [43,44] or relied on proteomic and metabolomic data from UVB irradiated tissue extracts [45], but did not assess the enzymatic activities *in situ*. To investigate the activation of G6PD upon UV stress in the epidermis we had to i) develop a model which allowed targeting specific or all cells in the epidermis with UV radiation; ii) establish a method to measure the activity in specific cells of the epidermis; iii) investigate the immediate and long-term effects of UVB on G6PD activity and iv) test whether a metabolism modulating drug can serve as proof of principle for our model and elucidate the potential of targeting epidermal metabolism.

### 4.1 Automated recognition of epidermal strata and individual cells and adaptation of G6PD activity measurements to epidermal tissue

Tissue FAXS technology is an automated tissue histocytometry technology that has been developed and improved for two decades [25]. As this approach has been successfully used to identify the activity of G6PD and other enzymes in tumor tissue, we adapted it for application on epidermis. We used a combination of nuclear density and contrast mapping to predict epidermis, its basal and suprabasal strata, and cell boundaries and confirmed specificity with established epidermal markers (K14 and K10) [46]. This technique will be useful in further automated imaging studies of the epidermis, as the strata can reliably be mapped even without immunostaining. Furthermore we show successful application of the method in an epidermal equivalent model [47,48].

We found that the activity of G6PD in unstressed skin and skin equivalent was highest at the epidermal granular layer. This pattern did, surprisingly, not correspond to the antibody staining pattern of G6PD in human skin, which showed a maximal staining in the basal epidermal layer. Thus, the first novel finding resulting from this study was KC-differentiation dependent increase in G6PD activity. The known factors regulating G6PD activity are numerous, and most likely post-translational mechanisms could explain the effect [13,27]. These include phosphorylation [49], glycosylation [50], multimerization and direct binding to proteins, and further studies will need to elucidate which of those act differentiation - dependently in the homeostatic epidermis.

One gradient along epidermal KC differentiation that requires NADPH - and thus active G6PD - is the UVB-protective glutathione barrier [7], as the pool of reduced Glutathione (GSH) is maintained via NADPH that serves as co-factor for glutathione reductase. This gradient provides the highest levels of reduced glutathione in the differentiated layers, while in basal KC antioxidant levels are lower to permit cell death upon irreparable UV damage. Our finding that steady state G6PD activity is highest in the granular layer of the epidermis is compatible with this concept.

### 4.2 Immediate and long-term effects of UVB on epidermal G6PD at cellular resolution

Solar radiation is the main source of extrinsic damage to the skin and causes damage to DNA, proteins and lipids either directly, through generation of photoproducts or indirectly through formation of ROS [[51], [52], [53], [54]]. Therefore several constitutive and inducible mechanisms for protection from oxidative damage exist in the skin. These include DNA damage repair [52], epidermal synthesis of antioxidant molecules [55], and of enzymes [7,56] which rely on GSH for recycling. GSH is also of high importance for the UV response as it directly reacts with UV-generated reactive species, including singlet oxygen hydroxyl radical and UV generated (lipid) aldehydes [57]. The pool of GSH thus needs to be replenished via NADPH after UV exposure. The enzymes G6PD and 6PGDH of the oxidative PPP are the main sources of NADPH in many tissues [58]. Our data localize, at cellular resolution, for the first time the rapid UVB inducible changes in G6PD activity within the epidermis that had previously been observed in cultured cells [11] and supports other findings on metabolic redesign in DDR [42]. Induction of activity was observed in basal, low- and high suprabasal cell populations, with the highest relative increase observed in the low suprabasal cells. Our histocytometric approach allowed another interesting finding, which was that those cells in the basal and low suprabasal layer that stained positive for γH2AX upon UVB irradiation did show the strongly increased G6PD activity, linking for the first time DNA damage repair and metabolic adaptation to UV in a tissue at cell resolution.

There have been several reports that might explain the mechanistic basis for such a connection between metabolic adaptations and repair of various types of UV-induced DNA damage in the skin. Firstly, long wavelength UVA exposure elevates glucose consumption, which is required for base excision repair (BER), and defective BER elevate glycolysis [59]. This may be due to regulation of glycolysis genes by the transcription factor Hif1 alpha. Defects in mitochondrial BER elevated ROS and thereby Hif1 alpha [60], which was also found elevated after UVB exposure [61]. In addition to these redox dependent mechanisms that can mainly be attributed to the contribution of longer wavelength UV light, also short wavelength UVB induced DNA damage can affect the PPP. DNA damage signaling via p53 can induce expression of the gene TP53-induced glycolysis and apoptosis regulator (TIGAR) and thereby activate the PPP [62]. UVB activates mechanistic target of rapamycin (mTOR) and downstream signaling [63] and mTORC1 activation again can mobilize not only glycolysis but also PPP [64]. Metabolization of glucose for re-supplementing nucleotides or nucleosides is further supported by the finding that glucose starvation limits DNA double strand break repair [65]. Interestingly, when NER or transcription coupled DNA repair are defective, glycolysis is inhibited and an overproduction of NADPH was observed in animal models and in skin fibroblasts from Cockayne syndrome patients [42]. The most likely mechanistic mediators are the ATM kinase and Hsp27, which are induced by UVB [34] and in turn bind to and activate G6PD [66,67]. Future studies will identify the feedback mechanisms between specific types of DNA damage, their recognition and repair on the one hand, and the G6PD system on the other hand. These further will elucidate the mechanistic base of rapid G6PD induction, its UV-dose or -fluence dependency, but also its inactivation at later timepoints in the UV response.

The observation on reduced G6PD activity in those KC that had been irradiated before being seeded into the epidermal equivalent was surprising, as one could have expected ongoing repair or adaptive sustained induction of this protective mechanism as has been observed also for the Nrf2 system, which facilitates NADPH regeneration and purine synthesis upon environmental stress [68]. Whether the reduced activity in those cells, which had not undergone cell death upon irradiation, reflects an early form of (photo)aging or senescence at the cellular level [69], will need to be investigated in the future. On that note, reduced G6PD activity was observed in geriatric KC isolated from aged donors [40].

### 4.3 Pharmacological modulation of epidermal G6PD activity

The drug metformin is the most widely used treatment for diabetes, and a modulator of gluconeogenesis and insulin sensitivity. In the skin, metformin prevents UVB induced skin tumorigenesis [41] and attenuates ROS mediated DNA damage exerted by the senescence-inducing drug paraquat [70], supposedly by interference with mitochondrial complex I activity and subsequent activation of AMP kinase. Numerous large scale ongoing studies investigate whether metformin would counteract age related decline also in non-diabetic healthy individuals [71]. Sun exposure, but also metabolic dysregulation affect skin aging [69,72], and conversely chronologically aged individuals display changed metabolism and redox responses. Thus, we tested whether we could modulate epidermal G6PD activity with metformin and observed a significant induction of activity 2 days post treatment, which was not observed seven days post treatment. At this time we can only speculate about the mechanism of induction, the most likely known pathway would involve AMPK activation/mitochondrial complex I inhibition, and in hepatocytes persistent activation of AMPK had been observed at comparable timepoints after metformin exposure [73]. To our knowledge, this is the first time that the ability of metformin to induce G6PD is shown in any tissue, and there are several angles where this ability might turn out useful in translation.

### 4.4 Potential applications of G6PD activity modulation in skin biology

Glucose levels are increased and PPP metabolites are decreased in aged skin, going in hand with a reduction of glycerolipid synthesis. Reduction especially of NADPH, a cofactor for synthesis of fatty acids [74] and other barrier lipid species (rev in Refs. [54]), could contribute to reduced barrier lipid recovery in the aged individuals [40,75,76]. Thus, modulating G6PD activity may be useful to counteract aging-induced functional decline of barrier recovery. Apart from diabetic skin complications [77], G6PD activation could protect also from oxidative and UV damage [78], and promote repair. Our approach will, as we hope be useful to investigate the contribution of the metabolic configuration of single, specialized cells within the skin and its appendages, to organ development, homeostasis and stress responses and aging.

## Declaration of competing interest

The authors have no conflicts of interest to state.
